# Jenner: A Hundred Years after

**Published:** 1896-05-16

**Authors:** 


### JENNER: A HUNDRED YEARS AFTER.

"Someone has blundered." On the 14th day of the current month exactly a hundred years had elapsed since the date of Jenner's first successful case of vaccination. America, Russia, and Germany are to celebrate the centenary of that event by demonstrations on a national and imperial scale. In England, so far as we know, no more notice will be taken of it than of the birth of an unknown pauper baby who may have happened to make his *début* in the world on that same day.

Two things may be said of vaccination at the present moment: First, it never was held in such high esteem as it now is among scientific medical men in every part of the world; and, secondly, it and the profession which advocates it were never so strong in the confidence of the enlightened portion of mankind as they now are. To these may, perhaps, be added a third affirmation, namely, that almost all the solid thought and sense of Great Britain at this moment are on the side of vaccination. The opposite side—the side of anti-vaccination—is the exclusive property of cranks, faddists, and exploiters of the ignorance and fanaticism of the least instructed among the people.

And yet, this is the time of all others which the medical profession in Great Britain has selected in which to honour the memory of Jenner with its supreme and absolute contempt. Among all our public bodies, medical societies, professional clubs and coteries, there is "none so poor," or shall we say, "so honourable as to do him reverence." It is sufficient to state the fact. No comment, however forcibly expressed, could make it look half so shameful or half so witless and stupid as it really is.

Everything written or spoken by the anti-vaccinators has always had our most careful consideration. But although we have tried to be scrupulously fair, we have never wavered from our conviction that vaccination is magnificently successful, and that as a matter of veritable and undeniable fact, proved in every part of the world, at all times, under all circumstances, and by all sorts and conditions of men, the prophylactic value of vaccination against small-pox is one of those certainties which are so firmly established that they never can be moved again so long as the world stands.

Jenner, in short, was one of the greatest benefactors which our race ever produced. This nation produced him; and at this moment, when the medical scientists of all nations are uniting as one man to do him honour, we do not hear that a single movement of any kind has been set on foot in this country for the exaltation of his name and fame among those whom the people should always be called upon to honour.

One body above all others should have placed itself at the head of a movement for the national celebration of Jenner's great achievement. That body is the Royal College of Physicians of London. Why, when all the scientific world was moving in this matter, has the College of Physicians stood nerveless and mute? Surely it should have taken advantage of this centenary to make amends for its past neglect, and should have placed itself at the head of the whole profession in a national movement to do honour to his memory.

But though the College of Physicians has covered itself with shame by its inertness in this matter, the great bulk of the profession, the general practitioners, who of all other persons in the world see most constantly and clearly the immeasurable value to the health of the world of Jenner's great discovery—these, we hold, are almost more blameworthy than even the College of Physicians. Why have the general practitioners of Great Britain taken no action on this memorable medical occasion? Why has the British Medical Association done nothing? Is there no public spirit among them—no *esprit de corps*—no regard for a medical Englishman, even though he be dead? We confess we cannot understand this condition of the medical mind. Even more difficult is it to understand the lack of generosity and of patriotism in the British public which allows an occasion such as this to slip by without a sign of appreciation for the great work done by their countryman. If British medicine should feel proud of Jenner, the British nation should be grateful as well as proud, for he has saved the lives of the people for a hundred years. Is it even yet too late for something to be done; for some responsible public body, or even private individual among us, to realise for itself or himself what vaccination has meant to the whole world for a hundred years? The scientific world is convinced that vaccination has meant the prevention of sufferings untold and the saving of millions of lives. Is the British public so indifferent to suffering and the loss of life that it has not a moment to spare for even a passing recognition of Jenner's memory and work? It would seem so! And the shame of it is inextinguishable.

